# Acceptability of policies targeting dietary behaviours and physical activity: a systematic review of tools and outcomes

**DOI:** 10.1093/eurpub/ckac053

**Published:** 2022-11-29

**Authors:** Marie Scheidmeir, Thomas Kubiak, Aleksandra Luszczynska, Janine Wendt, Daniel A Scheller, Biljana Meshkovska, Annabel Sandra Müller-Stierlin, Sarah Forberger, Karolina Łobczowska, Agnieszka Neumann-Podczaska, Katarzyna Wieczorowska-Tobis, Hajo Zeeb, Jürgen M Steinacker, Catherine B Woods, Jeroen Lakerveld

**Affiliations:** Department of Health Psychology, Johannes Gutenberg University, Mainz, Germany; Department of Health Psychology, Johannes Gutenberg University, Mainz, Germany; Department of Psychology, SWPS University of Social Sciences and Humanities, CARE-BEH Center for Applied Research on Health Behavior and Health, Wroclaw, Poland; Melbourne Centre for Behavior Change, Melbourne School of Psychological Sciences, University of Melbourne, Melbourne, VIC, Australia; Department of Internal Medicine, Sports- and Rehabilitation Medicine, University of Ulm, Ulm, Germany; Department of Internal Medicine, Sports- and Rehabilitation Medicine, University of Ulm, Ulm, Germany; Department of Nutrition, Institute of Basic Medical Sciences (UiO-PHN), University of Oslo, Oslo, Norway; Department of Psychiatry and Psychotherapy II, University of Ulm, Ulm, Germany; Department of Prevention and Evaluation, Leibniz Institute for Prevention Research and Epidemiology—BIPS, Bremen, Germany; Department of Psychology, SWPS University of Social Sciences and Humanities, CARE-BEH Center for Applied Research on Health Behavior and Health, Wroclaw, Poland; Deptartment of Palliative Care, Poznan University of Medical Sciences, Poznan, Poland; Deptartment of Palliative Care, Poznan University of Medical Sciences, Poznan, Poland; Department of Prevention and Evaluation, Leibniz Institute for Prevention Research and Epidemiology—BIPS, Bremen, Germany; Department of Internal Medicine, Sports- and Rehabilitation Medicine, University of Ulm, Ulm, Germany; Department of Physical Education and Sport Sciences, Physical Activity for Health, Health Research Institute, University of Limerick, Limerick, Ireland; Department of Epidemiology and Data Science, Amsterdam Public Health Research Institute, Amsterdam UMC, Vrije Universiteit Amsterdam, Amsterdam, the Netherlands; Upstream Team, www.upstreamteam.nl, Amsterdam UMC, Vrije Universiteit Amsterdam, Amsterdam, the Netherlands

## Abstract

**Background:**

Successful implementation of health policies require acceptance from the public and policy-makers. This review aimed to identify tools used to assess the acceptability of policies targeting physical activity and dietary behaviour, and examine if acceptability differs depending on characteristics of the policy and of the respondents.

**Methods:**

A systematic review (PROSPERO: CRD42021232326) was conducted using three databases (Science Direct, PubMed and Web of Science).

**Results:**

Of the initial 7780 hits, we included 48 eligible studies (*n* = 32 on dietary behaviour, *n* = 11 on physical activity and *n* = 5 on both), using qualitative and quantitative designs (*n* = 25 cross-sectional, quantitative; *n* = 15 qualitative; *n* = 5 randomized controlled trials; *n* = 3 mixed-methods design). Acceptability was analysed through online surveys (*n* = 24), interviews (*n* = 10), focus groups (*n* = 10), retrospective textual analysis (*n* = 3) and a taste-test experiment (*n* = 1). Notably, only 3 (out of 48) studies applied a theoretical foundation for their assessment. Less intrusive policies such as food labels and policies in a later stage of the implementation process received higher levels of acceptability. Women, older participants and respondents who rated policies as appropriate and effective showed the highest levels of acceptability.

**Conclusion:**

Highly intrusive policies such as taxations or restrictions are the least accepted when first implemented, but respondents’ confidence in the relevance and effectiveness of the policy may boost acceptability over the course of implementation. Studies using validated tools and a theoretical foundation are needed to further examine opportunities to increase acceptability.

## Introduction

Unhealthy dietary behaviours and a lack of physical activity have been recognized as the main risk factors contributing to the increasing prevalence of non-communicable diseases such as cardiovascular disease, diabetes and cancers.^1^ Therefore, policies targeting dietary behaviours and physical activity, such as the provision of information or measures that restrict choice by regulation are important levers for reducing the global burden of disease.^2^

For public health policies to be effective, it is critical that they are accepted by the public^3^ as well as policy-makers.^4^ Acceptability of policies can be defined as the perception among stakeholders that the implementation of a given policy is agreeable, palatable or satisfactory.^3^ While acceptability of policies has been studied in domains such as tobacco control and alcohol consumption, there is a need for further research on acceptability of policies in the domains of physical activity and diet.^5^ An older systematic review by Diepeveen *et al*.^5^ on public acceptability of government interventions examined various health behaviours, but diet and physical activity accounted only for a small proportion of policies analysed, and many have been studied and reported since.

Acceptability towards policies targeting dietary behaviours and physical activity may vary with characteristics of the policies and of the respondents (target population and/or policy-makers).^5^ According to the ‘Nuffield intervention ladder’, policies can be classified according to their degree of intrusiveness.^6^ Policies higher up the ladder are more intrusive (i.e. more restrictive) and are often perceived less acceptable as they tend to reduce one’s individual freedom. Acceptability is higher for less intrusive policies such as health media campaigns or warning labels.^5^ Acceptability can also vary with the stage of implementation,^5^ with policies in a later stage tending to be better accepted. Acceptability can be of key relevance across all stages of implementation: In an early stage, acceptability is crucial for the initial adoption, in an ongoing stage, it is needed for a successful integration into service settings. In a later stage, acceptability impacts the sustainability of a policy.^3^ Acceptability may also vary with characteristics of the target respondents. Diepeveen *et al*.^5^ found that women and older participants reported the highest levels of acceptability. The evaluation of acceptability can be studied in various ways but has been predominantly conducted through online surveys and questionnaires.^7^ Data on the validity and reliability of these measures are largely missing, with some exceptions, such as the Acceptability of Intervention Measure.^8^

The aim of this review was 2-fold: First, to identify tools used for assessing and evaluating acceptability of policies targeting physical activity and dietary behaviours. Secondly, to examine the acceptability of policies that relate to dietary and physical activity behaviours, and the role of policy and respondent characteristics on acceptability.

The present review was conducted within the Policy Evaluation Network (www.jpi-pen.eu).^9^ Since 2019, 28 European research institutions have collaborated in this network to advance knowledge on the effective implementation of policies and their impact in terms of improving health behaviours. In this review, we build on the previous work of Diepeveen *et al*.,^5^ extending that review to the focus areas of the Policy Evaluation Network by shifting the scope to dietary and physical activity behaviours to identify tools to analyse acceptability.

## Methods

We conducted a systematic review to identify and summarize (i) relevant literature on the acceptability of policies devised to target dietary and physical activity behaviours, and (ii) evaluation methods to assess acceptability. This review was conducted and reported according to the current PRISMA guidelines.^10^^,^^11^

### Search strategy

The primary search covered three databases: Science Direct, PubMed, Web of Science and Google Scholar. The search strategy included a range of behaviour-related keywords used in combination with policy (or related terms such as regulation, law, intervention) and keywords indicating the assessment of acceptability (e.g. support, opinion), which were customized to each database (see Supplementary material S1a: search strategy). We searched for studies published in English between 2010 and 2021 and reviewed bibliographies of the included studies to check for further relevant references.

### Study selection and inclusion criteria

We included original studies that explored acceptability towards a policy targeting dietary and/or physical activity behaviours. Studies were eligible if they investigated political acceptability, i.e. the perspective of individuals involved in the decision-making process and/or public acceptability, from the perspective of individuals potentially affected by a policy.^4^ We defined key terms in advance to facilitate and standardize the identification of studies (see Supplementary material S1b: inclusion criteria).

The inclusion of studies was decided upon in an iterative and hierarchical manner by two reviewers, starting out with the initial evaluation of study titles and the screening of abstracts, with full-text evaluations as the final step. The final inclusion of studies was based on two independent raters (A.S.M.-S., A.N.-P., D.A.S., J.W., K.L., K.W.-T., M.S. and S.F.) for each identified record, and any disagreements were discussed until consensus was reached.

#### Quality assessment

The quality of the included studies was assessed using the Critical Appraisals Skills Programme (CASP) for qualitative studies and randomized controlled trials,^12^ and the National Institutes of Health (NIH) Quality Assessment Tool for Observational Cohort and Cross-Sectional Studies.^13^ The quality appraisal was independently performed by two reviewers for each study (M.S., D.A.S., J.W., B.M. and K.L.), any disagreement was resolved by consensus.

#### Data extraction and synthesis

The following data were extracted from each study: study design, sample, data collection and analysis methods and results. Five categories of information were coded and extracted:

1. All types of measures used to gain information on acceptability;
2. Levels of acceptability;
3. Characteristics of target behaviour;
4. Characteristics of policies: the type and content of policies, the target population, intrusiveness of the intervention according to the Nuffield Intervention Ladder^6^ and stage of implementation (early, ongoing, late)^3^; and
5. Characteristics of target respondents: age, sex, country and socioeconomic status.

Two researchers independently extracted the data from included studies (M.S., B.M., J.W. and D.A.S.). Given the heterogeneity of the study methods and data, we opted for a narrative synthesis of the extraction outcomes. The synthesis was conducted by two researchers (T.K. and M.S.). Any disagreements during the data synthesis process were resolved by the consensus method.^14^ We mapped the included studies according to: (i) study type; (ii) data collection method; (iii) characteristics of policies; and (iv) characteristics of target respondents. Quantitative data synthesis followed the Synthesis without Meta-Analysis guidelines.^15^ To synthesize results on levels of acceptability, a vote counting on the direction of effect was conducted by two reviewers (M.S. and T.K.). Detailed vote counting results are included in Supplementary material S2.

## Results

**Figure 1:**
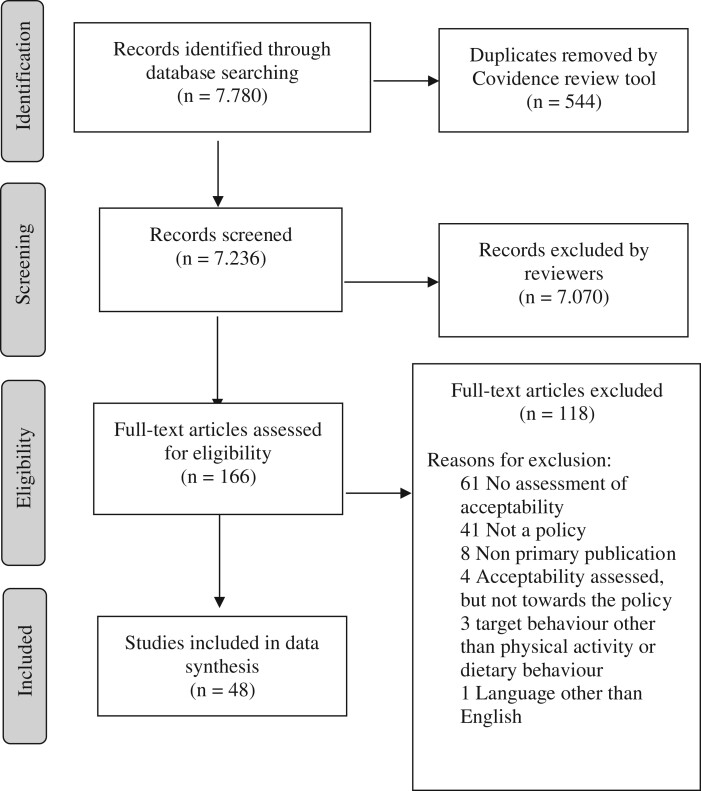
Flow diagram of studies included in the review

The PRISMA Flow chart illustrates the inclusion and exclusion of studies (figure 1). Our search identified a total of 7780 publications. Duplicates (*n* = 544) were automatically removed. A total of 7070 articles did not meet the inclusion criteria and were excluded during title and abstract screening. We examined the full texts of 166 publications; 49 studies met our inclusion criteria. One of these studies^16^ was excluded during data extraction, as the results section indicated an evaluation of appropriateness rather than acceptability (see Supplementary material S1b: exclusion criteria). The remaining 48 studies were included in our review. Table 1 gives an overview of study design, sample characteristics, target behaviours and data collection methods.

**Table 1:** Characteristics of the 48 included studies and key findings extracted for this review

Authors, year of publication	Study design	Target behaviour	Sample^a^	Acceptability theme^b^	Data collection method	Length^c^	Details on data collection provided by the authors
Aarts *et al*. (2011)^42^	Qualitative research	PA	Political: *N* = 25 policy officers	‘support’	Semi-structured face-to-face interviews	Ø 45 min	Open-ended questions about the current state municipalities regarding multi-sector policy action as well as opinions on facilitators and challenges for multi-sector policies. Support for the policy actions was determined based on the number of quotations in the transcripts (pooled for the four municipalities) about policies initiatives related to (the environmental determinants of) physical activity among children.
Alassaf *et al*. (2020)^34^	Cross-sectional study	Diet	Public: *N* = 1265	‘support’	Online survey	*N* = 1 item	One item rated on a two-point scale (‘yes’/‘no’): ‘Do you support the new policy mandating that restaurants display calories on their menus?’
Allender *et al*. (2012)^43^	Qualitative research	Diet and PA	Political: *N* = 11 (key informants, e.g. Chief Executive Officer of a local council; two healthy programme coordinators)	‘support’	Semi-structured interviews	*N* = 3 questions	The following information was asked from respondents: ‘Which interventions from the list of nine possible policy areas were likely to be supported within local government?’ Authors also asked participants for any other policy areas not included on the list that they felt might be relevant. Participants were asked to provide their view of the policy situation throughout the state of Victoria and not simply their own local council.
Beeken *et al*. (2013)^35^	Cross-sectional study	Diet	Public: *N* = 1986 (adults)	‘support’	Computer-assisted, face-to-face omnibus survey	*N* = 5 items	Participants were asked to rate their level of agreement for different statements such as: ‘The government should restrict advertising and marketing of unhealthy foods’.
Belizan *et al*. (2019)^56^	Mixed-method study	Diet and PA	Public: *N* = 250 (*n* = 206 survey; *n* = 44 interview)	‘acceptability’, no definition provided	(1) Semi-structured interviews, (2) Online survey	NA	Qualitative interviews used inductive inquiries consistent with the grounded theory approach.^71^ Themes identified: experience with implementing projects focusing on promoting physical activity and healthy diet; and key barriers and facilitators on designing, implementing and evaluating projects at the local level. Electronic survey contained both closed multiple response questions and open questions. Themes included were: experiences in projects related to the promotion of healthy habits over the last 5 years, barriers for projects ‘implementation, use of process and outcome measures for projects’ evaluation.
Bentley *et al*. (2015)^40^	Qualitative research	PA (SB)	Public: *N* = 24 (mothers of preschool children)	‘attitudes’	Semi-structured interviews	43 min (mean; min: 23, max: 64)	Interview questions on attitudes towards the specific targets of the guidelines. Data analysed thematically using a framework approach.^51^
Bhawra *et al*. (2018)^45^	Cross-sectional study	Diet	Public: *N* = 2729	‘support’	Online survey	*N* = 21 items (*n* = 1 item per policy)	For each policy, participants were asked, ‘Would you support or oppose a government policy that would require [policy]?’ and had the option of selecting, ‘Support’, ‘Neutral’, ‘Oppose’, or ‘Don’t Know’.
Bleich *et al*. (2010)^47^	Cross-sectional study	Diet	Public: *N* = 663	‘perception’	Telephone survey	*N* = 8 Items (*n* = 6 caloric labelling in chain restaurants; *n* = 2 calorie posting legislation)	Item example: ‘Do you favour or oppose the government requiring chain restaurants, such as McDonalds or Subway, to post calorie information on menus or menu boards for each food item at the point of purchase?’
Carson *et al*. (2014)^57^	Qualitative research	PA (SB)	Public: *N* = 27 (parents with a child under four years, who attended a child care centre)	‘perception’	Semi-structured focus groups	*N* = 6 questions	Questions: ‘What does sedentary behaviour mean to you? Is this definition similar/different than what you thought it was?’ ‘How clear are these guidelines?’ ‘What is your initial reaction to the guidelines? How do they make you feel as a parent (e.g. Irritated/Frustrated/Happy/Guilty)’ ‘Why do you think these guidelines are feasible/unfeasible or realistic/unrealistic?’ ‘The guideline sheet lists several health benefits for young children associated with meeting the guidelines. What are your thoughts about these statements? Do you agree or believe them?’ ‘From what source would you need this information from to consider it credible/trustworthy?’
Cradock *et al*. (2018)^58^	Cross-sectional study	PA	Public: *N* = 906	‘support’	Telephone survey	*N* = 5 items, (*n* = 1 item per policy)	Item example: ‘Do you support funding for programs that encourage walking and bicycling to school?’ Response format: ‘Yes’/’No’
Curbach *et al*. (2018)^59^	Cross-sectional study	PA	Public: *N* = 923 (delivery system actors: physicians and general practitioners)	‘attitudes’	Mail survey	*N* = 17 items	Questions focused on the respondents’ awareness and knowledge of the prevention scheme, prescribing behaviour, barriers to using the scheme, and on the physicians’ attitude towards patient counselling for physical activity and some socio-demographic items. Barrier items: For the barriers to using the scheme, the respondents could choose from a list of barriers to implementation and rate the relevance of these on a four-point Likert-scale.
Day *et al*. (2015)^60^	Qualitative research	Diet	Public: *N* = 128 (*n* = 117 pupils; *n* = 6 catering managers; *n* = 5 heads of schools)	‘perception’, authors use ‘acceptability’ synonymously (no definition provided)	Semi-structured focus groups	20–40 min	The focus group topic schedule used semi-structured open-ended questions to guide discussion, for example: ‘What do you think about your school meals? (healthiness/quality/choice/quantity?)’
De la Cruz-Góngora *et al*. (2017)^21^	Qualitative research	Diet	Public: *N* = 135	‘perception’, authors use ‘acceptability’ synonymously (no definition provided)	Focus groups	Ø 75 min	Focus groups following thematic analysis: Food label acceptability has been operationalized as ‘liking it and confidence in it’
Emm *et al*. (2013)^26^	Cross-sectional study	Diet and PA	Public: *N* = 188	‘support’	Online survey	*N* = 17 items (*n* = 7 for redistributive; *n* = 6 for compensatory and *n* = 4 for price-raising policies)	Participants were presented with descriptions of proposed obesity policies identified as having the largest potential impact on public health and which were politically feasible. Participants rated their agreement to the presented policy options via a seven-point Likert scale, ranging from 1 = ‘disagree strongly’ to 7 = ‘agree strongly’. The items were taken from a measure by Barry *et al*. (2009).^73^ Reliability for the subscales: redistributive: *α* = 0.71; compensatory: *α* = 0.52; price raising: *α* = 0.77
Faulkner *et al*. (2016)^41^	Qualitative research	PA (SB)	Public: *N* = 104 (*n* = 18 students; *n* = 52 parents; *n* = 17 teachers; *n* = 5 paediatricians; *n* = 16 qualified exercise professionals)	‘perception’, authors use ‘acceptability’ synonymously (no definition provided)	Semi-structured focus group interviews	NA	Question example: ‘In the current case for example, do people perceive a need for 24-hour movement guidelines and are the recommendations perceived as relevant, achievable, and acceptable to their lives as a parent, qualified exercise professional, or paediatrician?’
Fitzgerald *et al*. (2018)^61^	Qualitative research	Diet	Public: *N* = 13 (managing directors, owners, head chefs and franchises)	‘attitudes’	Semi-structured interviews	40–60 min	Interviews focused on participants’ perceptions and experiences, potential barriers and facilitators surrounding the implementation of calorie menu labelling. A semi-structured topic guide was developed to allow for comparisons of experiences and attitudes in relation to calorie menu labelling. The topic guides took an inductive approach and allowed for unique insights and perspectives to emerge. Framework approach^51^ was used for data analysis.
Gase *et al*. (2015)^48^	Cross-sectional study	PA (AT)	Public: *N* = 912 (registered voters in Los Angeles, CA, USA)	‘support’	Telephone survey	*N* = 31 items; 3–4 items per policy (*n* = 9)	Questions partially developed on already existing (Omnibus Household Survey, California Household Travel Survey), partially developed specifically for this study. Participants were asked whether they strongly supported, somewhat supported, somewhat opposed or strongly opposed ‘redirecting current federal, state or local transportation dollars’ to implement nine specific transportation improvements (increase traffic enforcement, develop more bike paths or lanes, etc.).
Hagmann *et al*. (2018)^17^	Cross-sectional study	Diet	Public: *N* = 5238 (participants in the Swiss food panel)	‘acceptability’, definition provided: ‘how individuals feel and think about the implementation or continued existence of policies’^2^	Online survey	*N* = 8 items	Respondents were provided with a short introductory statement: ‘Given the overweight problem (in our society), a lot of measures have been proposed (in order to tackle it).’ They were then asked to indicate their acceptance of different specific measures (‘How do you rate the following strategies to reduce sugar consumption in the Swiss population?’). Each item was rated on a seven-point response scale ranging from 1 (‘do not agree at all’) to 7 (‘fully agree’).
Joyce *et al*. (2020)^20^	Randomized cross-over trial	Diet	Public: *N* = 36 (school children, 6–9 years old)	‘acceptability’, no definition provided	Randomized cross-over trial	NA	A modified version of the United Stated Department of Agriculture, Food and Nutrition Services, Child Nutrition Programs, Team Nutrition try-day taste-testing ballot was used.^74^ Acceptability operationalized trough optics, taste fulfilment (satiation) and leftovers (plate waste weighing).
Julia *et al*. (2015)^25^	Cross-sectional study	Diet	Public: *N* = 1996	‘support’	Online survey	*N* = 7 items	Questionnaire items pertained to issues regarding acceptance of the tax (‘I support imposing a tax on sweetened beverages’, ‘I support imposing taxes on any and all foods and beverages which are bad for health’, ‘I support imposing a tax on sweetened beverages only if the money is then used to improve the health-care system’).
Jürkenbeck *et al*. (2020)^27^	Cross-sectional study	Diet	Public: *N* = 1035	‘support’	Online survey	*N* = 8 items	The participants had to evaluate statements about their general opinion regarding governmental interventions to support healthy eating behaviour, and statements about their own eating behaviour, using a five-point Likert scale ranging from + 2 (‘I totally agree’) to −2 (‘I do not agree at all’). Afterwards, various statements about specific policy interventions had to be evaluated on the same five-point Likert scale.
Kang *et al*. (2017)^46^	Qualitative study	Diet and PA	Public: *N* = 11715 (twitter users)	‘opinions’	Textual analysis (of Twitter data)	*N* = 480 tweets	Acceptability was defined through opinion mining on tweets. Tweets were categorized as either positive, neutral or negative. The opinions of tweets were extracted using an unsupervised, lexicon-based approach. After calculating both a positive and a negative value of each tweet, the method compares the two values to determine the polarity of the overall opinion and level of intensity in the tweet. The approach employs a number of look-up tables such as Linguistic Inquiry and Word Count.^72^
Kongats *et al*. (2019)^28^	Cross-sectional study	Diet	Public and political: *N* = 2702 (*n* = 2400 general public; *n* = 302 policy influencers)	‘support’, authors use ‘acceptability’ synonymously and refer to the review by Diepeveen et al.^4^	Online survey	*N* = 14 items	Participants were asked to rank their support for each policy via a four-point Likert scale. In total, 13 questions specific to policies promoting healthy eating were asked. The sample of policy influencers were asked to indicate their level of support for an additional 20 policy options on healthy eating. A binary variable for ‘support’ (combining strongly support with ‘somewhat support’ responses) vs. ‘opposition’ (combining ‘strongly oppose’ with ‘somewhat oppose’ responses) was created for the survey.
Kwon *et al*. (2019)^29^	Cross-sectional study	Diet	Public: *N* = 19857: (*n* = 3767 Australia, *n* = 3118 Canada, *n* = 4057 Mexico, *n* = 4047 UK, *n* = 4868 USA)	‘support’	Online survey	*N* = 13 items; (*n* = 1 item for each policy option)	Policy support measured via a three-point Likert scale. Item wording: ‘Would you support or oppose a government policy that would require…’ The scale consisted of ‘support’, ‘neutral’ and ‘oppose’. Additionally, participants could select ‘don't know’ or ‘refuse to answer’.
Le Roux *et al*. (2021)^62^	Cross-sectional study	Diet and PA	Public: *N* = 341 (students participating in the public health service programme)	‘satisfaction’	Paper survey	*N* = 14 items	‘Satisfaction questionnaire’, the scale consisted of ‘agree’ and ‘disagree’ or ‘yes’ and ‘no’.
Mathews *et al*. (2010)^39^	Cross-sectional study	PA	Political: *N* = 311 (*n* = 99 district officials; *n* = 215 principals)	‘attitudes’	Web-based and paper surveys	*N* = 18 items	‘School Travel Survey’ for district officials: http://prevention.sph.sc.edu/projects/Travel%20survey-District.pdf and school principals: http://prevention.sph.sc.edu/projects/Travel%20survey-Principals.pdf. Respondents were asked if they currently support local efforts to encourage walking to school and what type of impact walking can have on students’ health and academic performance.
Micheelsen *et al*. (2014)^63^	Randomized controlled trial	Diet	Public: *N* = 147 (*n* = 91 ‘New Nordic Diet’, *n* = 56 ‘Average Danish Diet’)	‘acceptability’, no definition provided	Question-naire	*N* = 14 items, 7 for each policy dimension (*n* = 2)	Acceptability measured as ‘practical acceptance’ and ‘eating acceptance’: The practical acceptance index consisted of all items in dimensions (1) ‘preparation of diet’ (e.g. ‘diet is easy to cook’) and (5) ‘continuation with the diet after the intervention’ (e.g. ‘It will not be too expensive to stay on my diet after the intervention’), together with one item from dimension (3) ‘everyday life and diet’ (e.g. ‘Following my diet does not require a lot of planning’). The eating acceptance index was made up of the remaining items in dimension (3) (e.g. ‘My diet fits nicely with my everyday life.’) along with all items in (2) ‘consumption and diet’ (e.g. ‘My diet is tasty’) and (4) ‘wellbeing and diet’ (e.g. ‘Eating my diet has given me energy’).
Milford *et al*. (2019)^44^	Mixed-methods study	Diet	Public: *N* = 2858 (*n* = 10 military kitchen staff; *n* = 2848 soldiers)	‘attitudes’	(1) Interviews and (2) paper-based question-naire	Interviews: NA Survey: *N* = 4 items	Qualitative interviews with kitchen staff on implementation of Meatless Monday: The topics the participants emphasized during the interviews were: relevance of targeting meat, knowledge, decision-making processes and soldiers’ attitudes. Quantitative survey on soldier attitudes to vegetarian food: ‘Reducing meat consumption is an efficient environmental measure’, ‘A high level of meat consumption is harmful to health’, ‘A well-balanced vegetarian diet contains all the nutrients the body needs’ and ‘Animal welfare is important to me’.
Morley *et al*. (2012)^30^	Cross-sectional study	Diet	Public: *N* = 1511	‘acceptability’, no definition provided	Phone Interviews	*N* = 18 items (*n* = 3 food labelling, *n* = 3 taxation, *n* = 1 product reformulation, *n* = 11 marketing)	Participants gave their opinions on food labelling, product reformulation, taxation and marketing through the use of Likert scales and close-ended questions. Participants were asked whether they were in favour of a number of policy practices and give their answer mostly using a five-point Likert scale. Some items instead used a three-point Likert scale (‘not regulate’/‘restrict’/‘stop this practice completely’). Some items also presented a selection of options from which participants were able to select their answer.
Nathan *et al*. (2011)^36^	Cross-sectional study	Diet	Public: *N* = 384 (delivery system actors: school principals and deputy assistant principals)	‘attitudes’	Computer Assisted Telephone Interview	20 min	Principals were asked to respond on a four-point Likert scale (1 = ‘strongly agree’ to 4 = ‘strongly disagree’), whether they felt that it is part of a school’s role to provide an environment which encourages healthy eating; that it is appropriate for schools to implement vegetable and fruit breaks and that vegetable and fruit breaks do not take away too much time from other educational priorities in the classroom or are disruptive to classroom routine.
Nguyen *et al*. (2015)^49^	Cross-sectional study	Diet	Public: *N* = 256 (educators)	‘perception’	Self-administered structured questio-nnaire	*N* = 3 items	Experts in the development of the food based dietary guidelines and primary-school-based interventions were involved in the development process of the questionnaire. The questionnaire was piloted at two schools and modified to enhance its validity before being used for the main study.
Odom *et al*. (2017)^23^	Cross-sectional study	Diet	Public: *N* = 7845 (2012: *n* = 3926; 2015: *n* = 3919)	‘attitudes’	Online survey	*N* = 6 items (*n* = 3 items per policy)	Questions on sodium in restaurants and manufactured foods: A five-point Likert scale was used to record responses: 1 = ‘strongly disagree’; 2 = ‘somewhat disagree’; 3 = ‘neither agree nor disagree’; 4 = ‘moderately agree’; and 5 = ‘strongly agree’, Responses of one, two and three were grouped together and termed ‘neutral/disagree’. Responses of four and five were grouped together and termed ‘agree’. Questions on sodium in school cafeterias, workplace cafeterias and quick-serve restaurants: A four-point Likert scale was used to record responses: 1 = ‘strongly oppose’; 2 = ‘slightly oppose’; 3 = ‘slightly support’; 4 = ‘strongly support’. Responses of one and two were grouped together and termed ‘neutral/not support’, and responses of three and four were collapsed into ‘support’.
Payán et al. (2017)^55^	Qualitative research	Diet	Public: *N* = 64 (high school students, 14-19 years old)	‘acceptability’, no definition provided	Focus groups	*N* = 6 questions	Focus group questions: ‘Where do you regularly eat lunch on a normal school day? Why?’ ‘About how often do you eat in your school cafeteria in a week? Why?’ ‘What types of changes have you noticed in your school cafeteria compared to previous years (if any)?’ ‘What do you think about these changes?’ ‘What do you like about your school’s cafeteria?’ [Probe: ‘What kinds of cafeteria foods do you like? Why?’] ‘What do you not like about your school’s cafeteria?’ [Probe: ‘What kinds of cafeteria foods do you not like? Why?’] ‘Describe ways to improve your school cafeteria and the meals served’.
Pell *et al*. (2019)^38^	Cross-sectional study	Diet	Public: *N* = 3104 (participants of International Food Policy Study)	‘support’	Online survey	*N* = 1 item	Single-item measure for support of the policy: ‘In 2018 a new sugary drink tax will be introduced in the UK. This aims to encourage manufacturers to reduce the sugar in drinks. The money will be spent on breakfast clubs, and sports in primary schools. Do you support or oppose this policy?’
Pettigrew *et al*. (2019)^24^	Cross-sectional study	Diet	Public: *N* = 607 (2008 survey; school staff members from 699 schools) *N* = 307 (2016 survey; school staff members from 798 schools)	‘attitudes’	Online survey	NA	Respondents were asked to report their attitudes to a range of potential policy extensions and their preferences for various forms of support that would enable them to effectively implement an enhanced version of the policy (rated on a five-point scale from ‘strongly disagree’ to ‘strongly agree’).
Regan *et al*. (2016)^31^	Cross-sectional study	Diet	Public *N* = 501	‘perspective’	Online survey	*N* = 13 items (one item for each policy)	Participants were asked to rate the level of importance that they felt the Irish government should assign to 13 different salt reduction policies. The assessed policies spanned government-industry cooperation, educational measures, restrictions on salt content of foods, labelling and fiscal measures. Each item was scored on a scale of one (‘not at all important’) to five (‘extremely important’).
Reynolds *et al*. (2019)^18^	Randomized controlled trial	Diet	Public: *N* = 7058	‘acceptability’ used, definition provided: ‘how individuals feel and think about the implementtation or continued existence of policies’.^2^	Online survey	*N* = 3 items	Acceptability of the policy was assessed using three items (*α* = 0.97; c.f^75^): ‘How acceptable do you find the policy?; How much are you in favour of the new policy being introduced?; Do you support or oppose the new policy?’. Each item was rated on a seven-point scale, labelled at either end (‘strongly oppose’; ‘strongly support’).
Riazi *et al*. (2017)^32^	Qualitative research	PA (SB)	Public: *N* = 10 (stakeholders: physicians) *N* = 92 (end users: parents)	‘impressions’, ‘acceptability’ used synonymously (no definition provided)	(1) Interview, (2) Focus groups	(1) 25–47 min (2) *N* = 6 discussion questions	(1) Interviews with stakeholders (experts in paediatric and family medicine, physical activity knowledge translation and research) and (2) Focus groups with end users (parents and early childhood educators). Both groups engaged in open-ended discussions about their first impressions of the Movement Guidelines, the clarity of the guidelines, and the need for an integrated guideline (e.g. ‘Do you find these integrated guidelines helpful or not helpful?’).
Richards *et al*. (2011)^65^	Qualitative research	PA (AT)	Public: *N* = 2784 (submissions from 16 councils)	‘support’	Textual analysis (of submissions to city council annual plans)	*N* = 2784 submissions	Analysis of submissions to city council annual plans related to active transport: All submissions were reviewed and categories of responses were created, for city, year, type of respondent, transport mode, what they were asking for and the reasons given for the request (e.g. health or sustainability).
Rida *et al*. (2019)^66^	Mixed-methods study	Diet	Public *N* = 15	‘attitudes’	Structured focus groups	*N* = 13 questions	Questions were developed to describe the nutrition knowledge of school food-personnel and the school food environment, contextualize attitudes and strategies of school food-personnel towards offering healthy school meals and barriers to face in offering and serving healthy meals. These questions were presented in two focus group sessions.
Robles *et al*. (2017)^37^	Cross-sectional study	Diet	Public: *N* = 1007	‘support’	Telephone survey	*N* = 9 items	Policy support was measured through a series of questions adapted from prior local obesity-related public opinion surveys carried out by Field Research in the Los Angeles region. Participants were questioned about their views on incentivizing/promotional policies and practices, limiting/restrictive policies and practices and changing business practices. All questions were close-ended. Participants selected their answers from a short list of possible alternatives.
Rydell *et al*. (2018)^67^	Randomized controlled trial	Diet	Public: *N* = 265 (participants eligible/nearly eligible for Supplemental Nutrition Assistance Program)	‘satisfaction’	Online survey	*N* = 4 items	Participants received a questionnaire with four close-ended questions to assess satisfaction with various elements of the programme via an anchored six-point Likert scale.
Signal *et al*. (2018)^22^	Qualitative research	Diet	Public and political: *N* = 22 (*n* = 3 politicians; *n* = 4 bureaucrats; *n* = 7 public health experts; *n* = 5 food industry leaders; *n* = 3 consumer representatives)	‘acceptability’, no definition provided	Key informant interview method^52^	*N* = 11 semi-structured interview questions	Question example: ‘Thinking about the next 5–10 years how acceptable is a New Zealand [tax/subsidy] likely to be to key stakeholders and why?’ Prompts: ‘Starting with (your sector). What about other key stakeholders like the public, politicians from various parties, the food industry, public health groups (e.g. Heart Foundation)’.
Swift *et al*. (2018)^68^	Qualitative research	Diet	Public: *N* = 412 (online forum users) *N* = 618 twitter accounts (*n* = 213 private accounts; *n* = 206 health-related organizations; *n* = 171 non-health-related organizations; *n* = 28 educational organizations)	‘perception’	Textual analysis (of online forum posts and tweets)	*N* = 412 (online forum posts) *N* = 618 (tweets)	Textual analysis of digital spaces: Study two examined posts on online forums about the Soft Drinks Industry Levy. Posts were categorized as either positive or negative. Study three examined tweets about the Sugar Smart app, likewise categorized as positive or negative.
Thomas-Meyer *et al*. (2017)^69^	Qualitative research	Diet	Public: *N* = 1158 online readers	‘support’	Textual analysis (of online comments on news articles)	*N* = 1645 comments	Thematic analysis of online reader comments using framework method.^51^ Analysis of public commenting on popular news websites in relation to SSB taxes. Comments were categorized as negative or positive, positive indicating support.
Turner-McGrievy *et al*. (2014)^70^	Cross-sectional study	Diet	Public: *N* = 71 (parents of children at a university-based childcare facility)	‘support’	Online survey	*N* = 37 items (referring to different aspects of the program)	Items assessing support for menu changes and support for adding more meatless entrée on a nine-point Likert Scale (1 being ‘not supportive’ and 9 being ‘supportive’). and open-ended questions to receive comments/opinions. Item example: ‘Below are the proposed nutrition standards for centres enrolled in South Carolina’s ABC program. Please rate your support for this change on a scale of 1 to 9 (1 being not supportive and 9 being supportive)’. In addition, parents were asked about their support for a centre-specific policy of only bringing healthy snacks for classroom celebrations.
Vargas-Meza *et al*. (2019)^19^	Randomized controlled trial	Diet	Public: *N* = 2105 (*n* = 697 Guideline Daily Allowances; *n* = 708 Warning Labels; *n* = 700 Multiple Traffic Lights)	‘acceptability’, based on theoretical framework by Grunert and Wills^76^	Online survey	*N* = 10 items (*n* = 3 likability; *n* = 4 attractiveness; *n* = 3 cognitive workload)	Label acceptability was evaluated using three indicators: likability, attractiveness and perceived cognitive workload, based on the framework of system acceptability by Nielsen.^50^ A questionnaire with ten statements was used to assess acceptability. Questions were answered by the participants using a five-point Likert scale.
Yun *et al*. (2018)^33^	Cross-sectional study	PA (SB and AT)	Public: *N* = 2519	‘support’	Online survey	NA	Aspects assessed: Individual responsibility for behaviours (e.g. providing programs to educate or motivate the general public about the importance of regular physical activity), modifying community environments (e.g. the quantity and quality of green spaces, safe areas for physical activity, and the design of neighbourhoods to encourage informal physical activity), targeting legislative changes to modify the environment (e.g. banning all traffic in high-use pedestrian areas during peak hours to support active or public transportation and restricting the use of elevators for trips to lower floors), focusing on economic levers (e.g. incentives, subsidies and tax credits).

a Political, any individuals involved in the decision-making process (e.g. policy-makers, politicians and informants from ministries); public, any individuals potentially affected by an SSBs tax (i.e. the public).

b Acceptability theme: aspects relating to acceptability in the study.

c Length refers to Item/Questions aiming at acceptability(-related concepts).

AT, active transport; Diet, dietary behaviour; NA, not available; PA, physical activity; SB, sedentary behaviour.

### Quality assessment results

Two randomized controlled trials were evaluated positively on seven and two on eight out of nine points on the CASP^12^ criteria. For the 15 qualitative studies, one was evaluated positively on seven, nine studies on eight and five studies on nine out of nine points of the CASP^12^ criteria. The most common points that were evaluated negatively were reflexivity and the consideration of the relationship between participants and researchers. All cross-sectional, quantitative studies received a fair (10 out of 25) or good (15 out of 25) quality rating.^13^ Based on the quality ratings, we decided to include all identified original studies into the review. Quality assessment results for each study are shown in Supplementary material S2. One recurring comment was the lack of a theoretical foundation for acceptability. Only three of the 48 studies used the term ‘acceptability’ and provided a clear definition.^17–19^

### Study characteristics

Table 2 gives an overview on study designs and data collection methods used in the included studies. Besides online surveys with varying response-formats and length, we identified qualitative approaches such as interviews, focus groups and retrospective textual analyses. Table 1 provides details on the specific data collection method for each study.

**Table 2:** Data collection methods across the study designs

Cross-sectional, quantitative design (*N* = 25)	Qualitative design (*N* = 15)	Mixed-method design (*N* = 3)	RCT^a^ (*N* = 5)
Online survey (*n* = 15), Paper questionnaire (*n* = 5), Mail survey (*n* = 2), Computer-assisted, face-to-face omnibus survey (*n* = 1), Telephone survey (*n* = 1), Computer Assisted Telephone Interview (*n* = 1)	Semi-structured Interviews (*n* = 6), Focus groups (*n* = 5), Retrospective textual analysis (*n* = 3), Combination of focus groups and interviews (*n* = 1)	Interviews and paper-based questionnaire (*n* = 1), Semi-structured interviews and online surveys (*n* = 1), Online survey and structured focus groups (*n* = 1)	Paper questionnaire (*n* = 1), Online survey (*n* = 3), Modified version of the nutrition try-day taste-testing ballot^74^ (*n* = 1)

a RCT, randomized controlled trials (*n* = 4), randomized cross-over trial (*n* = 1).

**Table 3:** Stage of implementation across studies by behavioural domain

Stage of implementation	Total^a^	Diet	PA	Diet and PA
Early stage (agenda setting)	23	13	4	2
Mid (ongoing) stage	17	6	5	3
Late stage (application)	13	10	4	–

a Total count: 53, three studies^33^^,^^45^^,^^47^ included policies in different stages of implementation.

Diet, dietary behaviour; PA, physical activity.

The following aspects were analysed for the studies following quantitative designs: ‘support’ (16/33), ‘perceptions’ (2/33), ‘attitudes’ (8/33) or ‘satisfaction’ (2/33), which relate to acceptability according to the Proctor’s definition.^3^ One randomized controlled trial evaluated food labelling acceptability through three indicators (likability, attractiveness and perceived cognitive workload).^19^ In one randomized cross-over trial, acceptability of a dietary guideline was explored through the assessment of optics, taste fulfilment (satiation) and leftovers (plate waste weighing).^20^

In qualitative studies, the following acceptability-related themes were explored: ‘attitudes’ (5/15) ‘views’ (3/15), ‘support’ (2/15), ‘impressions’ (1/15) and ‘perceptions’ (4/15).

### Policy and target group characteristics

Across the included studies, 32 addressed dietary behaviours, 11 physical activity and 5 a combination of both. Most of the policies addressing dietary behaviour aimed at a reduced calorie intake, mostly by lowering the consumption of foods high in sugar and fats. Policies addressing physical activity aimed at preventing sedentary behaviours or promoting active forms of transportation such as walking or cycling. A detailed description of the behaviours targeted in each study is available in Supplementary material S3.

#### Characteristics of policies included in the review

Of the 48 included studies, 17 measured acceptability for more than one policy, comparing, for example, different policies in one policy area (e.g. three different food labels)^21^ or assessing acceptability of different types of policies (e.g. campaigns, labels and restrictions).^22^ For detailed information on policy type and content see Supplementary material S3.

##### Stage of implementation

We classified policy implementation stage according to Proctor *et al*.^3^ into early stage (agenda setting), mid-(ongoing) stage and late stage (application) (Table 3).

Results from three studies^23–25^ showed that policies in a later stage received more support from both policy-makers and the target population than in earlier stages. A study conducted by Pettigrew *et al*.^24^ compared support among school staff members for a school-food policy between 2008 and 2016 and found significantly higher levels of support in 2016 (2008: 77%, 2016: 83%; *P* < 0.01; Cohen’s *d*: 0.22). Support for sodium limitation policies increased significantly from 2012 to 2015 for school canteens (2012: 80.0%, 2015: 84.9%; *P* < 0.05), workplace cafeterias (2012: 71.2%, 2015: 76.6%; *P* < 0.05) and quick serve restaurants (2012: 70.8%, 2015: 76.7%; *P* < 0.05),^23^ and a soda taxation policy in France received medium-high levels of support 3 years after its implementation (2012: 48.2; 2015: 48.2–72.5%; *P* < 0.05).^25^

##### Level of intrusiveness

We rated policies according to the steps in the Nuffield ladder^6^ and divided them into three groups: Highly intrusive policies (guiding choice through disincentives and eliminating/restricting choice, *n* = 25), moderately intrusive policies (guiding choice through incentives and by changing the default policy, *n* = 23) and low intrusiveness (providing information and enabling choice, *n* = 29).

Eleven studies reported a tendency that highly intrusive policies received less support than less intrusive policies.^17^^,^^18^^,^^22^^,^^26–33^ For instance, Kwon *et al*.^29^ found lower levels of support for a marketing ban on unhealthy foods (34.7%) than for subsidies on fruit and vegetables (68.2%).

A total of 15 studies investigated policies aimed at (school-)children, 10 of which were categorized as low and 5 as moderately intrusive. The policies with low levels of intrusiveness (*n* = 10) received predominantly high levels of acceptability (8/10). For example, 86% support was found for a policy promoting active school transport,^39^ while all moderately intrusive policies (*n* = 5) received mixed (3/5) or low (2/5) levels of acceptability.

##### Levels of feasibility, appropriateness and perceived effectiveness

Ten studies analysed appropriateness, feasibility or perceived effectiveness as implementation outcomes in addition to acceptability.^20^^,^^28^^,^^30^^,^^34–41^ Six studies^28^^,^^30^^,^^34–37^ measured appropriateness, such as the perceived importance or relevance of the policy.^3^ Findings showed that target group respondents who strongly believed that obesity was a serious problem were overall more supportive of healthy nutrition policies [beta = 0.36, 95% confidence interval (CI) 0.24–0.47].^37^

Three studies explored feasibility alongside acceptability.^20^^,^^29^^,^^41^ Feasibility is defined as the extent to which a policy can be successfully used or carried out within a given setting.^3^ Within each of these studies, policies were perceived equally acceptable and feasible. All studies that reported on perceived effectiveness,^34^^,^^38–40^ found that policies which were rated highly effective, also received high levels of acceptability. For example, Pell *et al*.,^38^ found similarly high levels for acceptability (70%) and perceived effectiveness (71%) for a sugary drink tax in the UK.

#### Characteristics of respondents

A majority of the included studies investigated public acceptability among the policies’ target populations (42/48). Three studies investigated political acceptability of policies (e.g. in samples that consisted of policy-makers),^39^^,^^42^^,^^43^ the remaining two studies investigated both political and public acceptability.^22^^,^^28^ The primary studies were predominantly from North America (18/48) and Europe (18/48), the remaining studies originated from Central and South America (6/48) and Oceania (6/48). Table 1 provides an overview on sample characteristics for each study. For further details on samples including country of origin, see Supplementary material S3.

##### Political vs. public acceptability

Four studies identified a lack of acceptability as a significant barrier to implementation^28^^,^^40^^,^^41^^,^^44^ and high acceptability as a facilitator among policy-makers, for the implementation of policies.^39^ One study reported higher levels of support from policy influencers (80%) than from the public (63.3%) towards healthy eating policies.^28^

##### Gender, age and socioeconomic status of respondents

Ten studies reported gender differences, with women generally showing higher levels of acceptance towards policies regardless of target behaviour or policy type.^17^^,^^18^^,^^25^^,^^29^^,^^33^^,^^35^^,^^37^^,^^45–47^

Moreover, three studies found that compared to male target respondents, women showed a tendency to be more supportive of more intrusive policies such as salt limits,^45^ restrictions to limit access to high-fat foods,^35^ high sugar drinks^17^ or restrictions to ban traffic in certain areas to facilitate active travel.^33^ Five studies found a link between acceptability and age.^25^^,^^31^^,^^33^^,^^45^^,^^48^ Four out of five studies found the tendency that older participants reported relatively higher levels of acceptability than younger participants, especially for highly intrusive policies such as marketing bans for unhealthy foods.^25^^,^^31^^,^^33^^,^^45^ Julia *et al*.,^25^ showed that older participants were more likely to support a soda tax than younger counterparts [odds ratio (OR) = 2.37; 95% CI 1.60, 3.49 for >65 years old vs. 26–45 years old; *P* < 0.001]. One study showed an opposite effect for a policy promoting active transportation: Levels of support were lower among those over age 65, when compared with those in the youngest age category (18–24 years).^48^

Six studies reported differences in acceptability according to socioeconomic status of the policy target population,^18^^,^^25^^,^^30^^,^^33^^,^^35^^,^^49^ i.e. participants with a higher educational background rated policies to change product sizes as more acceptable than those with low education (OR = 0.31; 95% CI 0.19–0.52; *P* < 0.001).^18^ Three studies found that taxation policies were more acceptable among lower socioeconomic groups, if they were combined with another policy action.^22^^,^^25^^,^^45^ In a study conducted by Julia *et al*.,^25^ 22.4% more participants reported that they would support a taxation policy, if combined with a subsidy to fund healthcare system improvements (48.5% vs. 72.7%).

## Discussion

We set out to identify tools used to assess acceptability of policies targeting diet and physical activity, and to examine acceptability with regard to characteristics of the various policies. We found a wide range of different approaches and tools to study this topic.

Few studies presented a link to an existing framework or a theoretical foundation of acceptability. Two papers^17^^,^^18^ defined acceptability according to the definition provided by Sekhon *et al*.^7^ and one paper^19^ based their assessment of acceptability on the framework of system acceptability by Nielsen (1993).^50^ Moreover, most of the measures did not include information on the reliability and validity of the measures. Only 2^26^^,^^50^ of the 38 survey studies reported on reliability. In contrast, most qualitative approaches applied a theoretical foundation to their measures such as the framework method^51^ or key informant interview technique.^52^

### Policy characteristics

More than three quarters (32/48) of the studies included in this review analysed acceptability of policies targeting dietary behaviours, while findings including policies targeting physical activity were relatively scarce (16/48). Although policies targeting physical activity were well accepted in the included studies, there is a gap in research on the acceptability of policies targeting physical activity. This needs to be addressed by future research, as such policies have the potential to facilitate numerous health benefits including a reduction in coronary heart disease, stroke and diabetes.^1^

Our findings on acceptability according to stage of implementation and intrusiveness are in line with existing literature for other health behaviours^3^^,^^5^: highly intrusive policies were generally less accepted, especially by men, younger age groups and those with a lower educational background, but acceptability by policy-makers and the target groups of the policies may increase over time. With highly intrusive policies, which are often more effective than less intrusive policies,^6^ governments need stronger justification for implementation in order to achieve sufficient levels of acceptability. This is substantiated by our findings on the relationship between acceptability and other implementation outcomes: Respondents who rated the policies to be effective, appropriate or feasible also found them to be acceptable.^20^^,^^28^^,^^30^^,^^34–41^

### Target group characteristics

Our findings on acceptability with regard to sex, age and socioeconomic status and were largely consistent with previous research. The tendency that women are more likely to support intrusive policies has been shown for other health-related behaviours such as smoking and alcohol consumption.^5^ A reason for the observed gender difference may lie in the domain of dietary behaviours, as women are more often subject to weight discrimination and may be sensitive and supportive to diet-related policies.^53^ We were also able to replicate the finding of Diepeveen *et al*.,^5^ that acceptability seems to increase with age. This was particularly true for highly intrusive policies.^17^^,^^35^^,45^ An exception was a study conducted by Gase *et al*., which found higher support among younger participants towards a policy re-directing transportation funds to active transport than among older participants.^48^ This may be due to younger individuals more often relying on alternatives to cars as means of transportation, such as bicycles.^54^ Six studies reported on levels of acceptability varying with socioeconomic status of the respondents.^18^^,^^25^^,^^30^^,^^33^^,^^35^^,49^ A majority found less support among groups with lower socioeconomic status for pricing and taxation policies,^25^^,^^30^^,^^33^^,^^35^^,49^ possibly because they were economically more affected that individuals with higher income. Moreover, we found that taxation policies were more popular among lower socioeconomic groups, when they were combined with a subsidy,^25^^,45^ e.g. for produce.^22^ This information may be useful when considering taxation policies in the future. Overall, we observed a lack of research addressing equity- and diversity-related factors: Samples were predominantly White, highly educated, and from middle- to high-income countries such as the USA and Europe. Few studies mentioned this as a limiting factor and fewer drew conclusions on the acceptability of policies in minority groups.^29^^,55^

### Strengths and limitations of the current review

To our knowledge, this is the first systematic review to comprehensively map evidence on public and political acceptability focusing on dietary and physical activity behaviours. We identified a variety of tools to measure acceptability and were able to identify gaps in the existing research and ways to strengthen future studies. There are limitations to our findings: Because of the heterogeneous designs, the large variety in assessment methods and heterogeneous answer formats, data of our literature search were not well suited for quantitative synthesis via meta-analytic procedures.

Another weakness of our review is the limited search range. We restricted ourselves to searching electronic, scientific databases excluding grey literature and political documents. This may be a reason why research and findings on political acceptability may be not well represented in our studies.

### Implications for policy-makers

Our findings show that intrusive policies are generally less well accepted, but become more accepted with time. This needs to be considered when facing decisions to implement particularly unpopular yet potentially highly effective policies. Over time, people may get used to the measures implemented by governments and become more accepting of such policies. Moreover, findings on appropriateness indicate that people who recognize the relevance of policies, e.g. because they are aware of the risks of certain behaviours, show a higher support of government action. Therefore, it is crucial that policy-makers provide well-communicated justifications for policies.

### Implications for research on policy acceptability

In order to monitor acceptability reliably both from a political and public perspective, we would recommend further research to consider three steps to increase quality and meaningfulness of publications:

1. The use of a pre-set definition of acceptability and further included terms and outcomes based on scientific theories/models, such as the framework provided by Proctor *et al*.^3^
2. The use of reliable, validated tools based on a framework such as the Acceptability of Intervention Measure.^8^
3. The inclusion of more diverse samples in terms of socioeconomic status, ethnicities and cultural backgrounds to gain a more holistic societal perspective.

Since we found a notable imbalance in target groups (public vs. political) and behaviours (dietary behaviour vs. physical activity), further research should aim at including the political perspective more broadly and to expand research activities on acceptability towards policies targeting physical activity.

## Conclusions

Highly intrusive policies such as taxations or restrictions are the least accepted when first implemented, but respondents’ confidence in the relevance and effectiveness of the policy may boost acceptability over the course of implementation. The current evidence is mostly based on studies done in European and North American contexts. Studies using validated tools and a theoretical foundation are needed, to further examine opportunities to increase acceptability.

## Supplementary data

Supplementary data are available at *EURPUB* online.

## Funding

The PEN project (www.jpi-pen.eu) is funded by the Joint Programming Initiative ‘A Healthy Diet for a Healthy Life’ (JPI HDHL), a research and innovation initiative of EU member states and associated countries. The funding agencies supporting this work are (in alphabetical order of participating countries): France: Institut National de la Recherche Agronomique (INRA); Germany: Federal Ministry of Education and Research (BMBF), grant no. FKZ: 01EA1818D/PEN70/03/2018; Ireland: Health Research Board (HRB); Italy: Ministry of Education, University and Research (MIUR); The Netherlands: The Netherlands Organisation for Health Research and Development (ZonMw); New Zealand: The University of Auckland, School of Population Health; Norway: The Research Council of Norway (RCN); Poland: The National Centre for Research and Development (NCBR), grant no. JFA PEN/I/PEN44/03/2018.

*Conflicts of interest*: None declared.

Key points

- Incoherent definitions, lack of theoretical frameworks and limited psychometric information characterizes policy acceptability tools.
- Highly intrusive policies are less accepted, but acceptability may increase over time of policy implementation.
- Acceptability is rated higher when the policy is perceived to be highly appropriate and effective.
- Economic policies are more acceptable to groups with lower socioeconomic status when combined with a subsidy.
- Women are more likely than men to report public health policies as acceptable, irrespective of behaviour or policy type.

## Supplementary Material

ckac053_Supplementary_Data
