# Supplementary material for: Acceptability of policies targeting dietary behaviours and physical activity: a systematic review of tools and outcomes
Source: Eur J Public Health. 2022 Nov 29;32(Suppl 4):iv32–49. doi: 10.1093/eurpub/ckac053 (PMC9897019; doi:10.1093/eurpub/ckac053)
Supplement: ckac053_Supplementary_Data [file ckac053_supplementary_data.zip › ckac053_Supplementary_Data/Scheidmeir_Acceptability_SuppleMat1a.pdf]

## Supplement 1a: Search strategy

Supplementary Table S1a
Full list of 3 groups of keywords applied in the searching strategy

1. **target behaviour:** (physical activity[tiab] OR exercise[mh] OR sport [mh] OR fitness [tiab] OR run* [tiab] OR move* [tiab] OR cycle[tiab] OR sedentary [mh] OR television*[mh] OR console [tiab] OR gaming [tiab] OR inact* [tiab] OR low energy expen*[tiab] OR diet*[mh] OR nutrition [mh] OR fat*[tiab] OR sugar*[tiab] OR sweetened[tiab] OR salt[tiab] OR sodium[tiab] OR meal[tiab] OR weight*[mh] OR overweight*[mh] OR obes*[mh] OR calorie[mh])

2. **acceptability:** (opinion [mh] OR attitude [mh] OR accept*[tiab] OR psychosocial factors[tiab] OR approv*[tiab] OR endors*[tiab] OR support[mh] OR barrier[mh] OR persua*[tiab] OR convince [tiab] OR agree*[mh] OR oppose*[tiab] OR opinion*[mh] OR favour*[tiab])

3. **the type of action:** (polic*[mh]) OR program*[mh] OR strateg*[mh] OR practic[tiab] OR government[mh] OR law [mh] OR regulation*[tiab]) OR intervention[mh] OR soft law[tiab] OR action plan[tiab] OR action program*[tiab] OR national stratgy[tiab] OR guideline*[mh])

Applied limiters: Publication dates: 2010-2021, English language only.
