# Supplementary material for: Acceptability of policies targeting dietary behaviours and physical activity: a systematic review of tools and outcomes
Source: Eur J Public Health. 2022 Nov 29;32(Suppl 4):iv32–49. doi: 10.1093/eurpub/ckac053 (PMC9897019; doi:10.1093/eurpub/ckac053)
Supplement: ckac053_Supplementary_Data [file ckac053_supplementary_data.zip › ckac053_Supplementary_Data/Scheidmeir_Acceptability_SuppleMat4.docx]

**Supplement 4: Additional references**

41 Faulkner G, White L, Riazi N, Latimer-Cheung AE, Tremblay MS. Canadian 24-Hour Movement Guidelines for Children and Youth: Exploring the perceptions of stakeholders regarding their acceptability, barriers to uptake, and dissemination. Appl Physiol Nutr Metab Physiol Appl Nutr Metab 2016;41:S303-310.

42 Aarts MJ, Jeurissen MPJ, van Oers HAM, Schuit AJ, van de Goor IAM. Multi-sector policy action to create activity-friendly environments for children: a multiple-case study. Health Policy Amst Neth 2011;101:11–19.

43 Allender S, Gleeson E, Crammond B, et al. Policy change to create supportive environments for physical activity and healthy eating: which options are the most realistic for local government? Health Promot Int 2012;27:261–74.

44 Milford AB, Kildal C. Meat Reduction by Force: The Case of “Meatless Monday” in the Norwegian Armed Forces. Sustainablility 2019;11. doi:10.3390/su11102741.

45 Bhawra J, Reid JL, White CM, Vanderlee L, Raine K, Hammond D. Are young Canadians supportive of proposed nutrition policies and regulations? An overview of policy support and the impact of socio-demographic factors on public opinion. Can J Public Health 2018;109:498–505.

46 Kang Y, Wang Y, Zhang D, Zhou L. The public’s opinions on a new school meals policy for childhood obesity prevention in the U.S.: A social media analytics approach. Int J Med Inf 2017;103:83–88.

47 Bleich SN, Pollack KM. The publics’ understanding of daily caloric recommendations and their perceptions of calorie posting in chain restaurants. BMC Public Health 2010;10:121.

48 Gase LN, Barragan NC, Simon PA, Jackson RJ, Kuo T. Public awareness of and support for infrastructure changes designed to increase walking and biking in Los Angeles County. Prev Med 2015;72:70–75.

49 Nguyen KA, de Villiers A, Fourie JM, Bourne LT, Hendricks MK. The feasibility of implementing food-based dietary guidelines in the South African primary-school curriculum. Public Health Nutr 2015;18:167–75.

50 Nielsen J. Usability engineering. Boston: Academic Press, 1993.

51 Gale NK, Heath G, Cameron E, Rashid S, Redwood S. Using the framework method for the analysis of qualitative data in multi-disciplinary health research. BMC Med Res Methodol 2013;13:117.

52 Tremblay MA. The Key Informant Technique: A Nonethnographic Application. Am Anthropol 1957; 59:688-701.

53 Puhl RM, Heuer CA. Obesity Stigma: Important Considerations for Public Health. Am. J. PUBLIC Health. 2010;100:1019-28.

54 O׳Hern S, Oxley J. Understanding travel patterns to support safe active transport for older adults. J Transp Health 2015;2:79-85.

55 Payan DD, Sloane DC, Illum J, Farris T, Lewis LB. Perceived Barriers and Facilitators to Healthy Eating and School Lunch Meals among Adolescents: A Qualitative Study. Am J Health Behav 2017;41:661-69.

56 Belizan M, Chaparro RM, Santero M, et al. Barriers and Facilitators for the Implementation and Evaluation of Community-Based Interventions to Promote Physical Activity and Healthy Diet: A Mixed Methods Study in Argentina. Int J Environ Res Public Health 2019;16. doi:10.3390/ijerph16020213.

57 Carson V, Clark M, Berry T, Holt NL, Latimer-Cheung AE. A qualitative examination of the perceptions of parents on the Canadian Sedentary Behaviour Guidelines for the early years. Int J Behav Nutr Phys Act 2014;11:65.

58 Cradock AL, Barrett JL, Chriqui JF, et al. Driven to Support: Individual- and County-Level Factors Associated With Public Support for Active Transportation Policies. Am J Health Promot AJHP 2018;32:657-66.

59 Curbach J, Apfelbacher C, Knoll A, Herrmann S, Szagun B, Loss J. Physicians’ perspectives on implementing the prevention scheme ‘Physical Activity on Prescription’: Results of a survey in Bavaria. Z Evidenz Fortbild Qual Im Gesundheitswesen 2018;131-132:66-72.

60 Day RE, Sahota P, Christian MS, Cocks K. A qualitative study exploring pupil and school staff perceptions of school meal provision in England. Br J Nutr 2015;114:1504-14.

61 Fitzgerald S, Gilgan L, McCarthy M, Perry IJ, Geaney F. An evaluation and exploration of Irish food-service businesses’ uptake of and attitudes towards a voluntary government-led menu energy (calorie) labelling initiative. Public Health Nutr 2018;21:3178-91.

62 Le Roux E, Muro MM, Mognon K, et al. A governmental program to encourage medical students to deliver primary prevention: experiment and evaluation in a French faculty of medicine. BMC Med Educ 2021;21.

63 Micheelsen A, Havn L, Poulsen SK, Larsen TM, Holm L. The acceptability of the New Nordic Diet by participants in a controlled six-month dietary intervention. Food Qual Prefer 2014;36:20-26.

64 Nathan N, Wolfenden L, Butler M, et al. Vegetable and fruit breaks in Australian primary schools: prevalence, attitudes, barriers and implementation strategies. Health Educ Res 2011;26:722-31.

65 Richards R, Murdoch L, Reeder AI, Amun Q. Political activity for physical activity: health advocacy for active transport. Int J Behav Nutr Phys Act 2011;8:52.

66 Rida Z, Hall E, Hasnin S, Coffey J, Dev DA. The perception of school food-service professionals on the implementation of the Healthy, Hunger-Free Kids Act of 2010: a mixed-methods study. Public Health Nutr 2019;22:1960-70.

67 Rydell SA, Turner RM, Lasswell TA, et al. Participant Satisfaction with a Food Benefit Program with Restrictions and Incentives. J Acad Nutr Diet 2018;118:294–300.

68 Swift JA, Strathearn L, Morris A, Chi Y, Townsend T, Pearce J. Public health strategies to reduce sugar intake in the UK: An exploration of public perceptions using digital spaces. Nutr Bull 2018;43:238-47.

69 Thomas-Meyer M, Mytton O, Adams J. Public responses to proposals for a tax on sugar-sweetened beverages: A thematic analysis of online reader comments posted on major UK news websites. PloS One 2017;12.

70 Turner-McGrievy GM, Hales SB, Baum AC. Transitioning to new child-care nutrition policies: nutrient content of preschool menus differs by presence of vegetarian main entrée. J Acad Nutr Diet 2014;114:117-23.

71 Glaser B, Strauss A. The Discovery of Grounded Theory: Strategies for Qualitative Research. 1st ed. Abingdon, UK: Routledge, 2009.

72 Pennebaker JW, Francis ME, Booth RJ. Linguistic Inquiry Word Count: LIWC. Mahwah, US: Lawrence Erlbaum Associates, 2001.

73 Barry CL, Brescoll VL, Brownell KD, Schlesinger M. Obesity metaphors: How beliefs about the causes of obesity affect support for public policy. Milbank Q 2009;87, 7-47.

74 USDA Food and Nutrition Service. Taste Test Ballot [Internet]. USDA-FNS, USA [cited 2021Dec15]. Available from <https://www.fns.usda.gov/sites/default/files/tn/TNevents_appendixrepro1.pdf>

75 Reynolds JP, Pilling M, Marteau TM. Communicating quantitative evidence of policy effectiveness and support for the policy: three experimental studies. Soc Sci Med 2018a;218, 1-12.

76 Grunert KG, Wills JM. A Review of European Research on Consumer Response to Nutrition Information on Food Labels. J Public Health 2007;15, 385-399.
