# Supplementary material for: Acceptability of policies targeting dietary behaviours and physical activity: a systematic review of tools and outcomes
Source: Eur J Public Health. 2022 Nov 29;32(Suppl 4):iv32–49. doi: 10.1093/eurpub/ckac053 (PMC9897019; doi:10.1093/eurpub/ckac053)
Supplement: ckac053_Supplementary_Data [file ckac053_supplementary_data.zip › ckac053_Supplementary_Data/Scheidmeir_Acceptability_SupplMat1b.docx]

**Supplement 1b: Inclusion and exclusion criteria**

Inclusion criteria:

1. study is measuring acceptability of a policy targeting diet and/or physical activity behaviours (including sedentary behaviours)

2. the population of the study is composed of any individuals involved in the decision-making process (e.g. policy-makers, politicians and officials of ministries) or any individuals potentially affected by a policy targeting PA/diet (i.e. the public), (c.f. ^1^)

Definitions of included concepts:

Policies targeting a) diet or b) physical activity:

(a) Policies are purposeful decisions, plans and actions made by voluntary or authoritative actors in a system designed to create system-level change to directly or indirectly achieve specific societal goals. Within this definition, public policy is a form of government action usually expressed in a law, a regulation, or an order.^2^

Following definitions of acceptability are included:

(b) Public acceptability of policies i.e. how individuals feel and think about the implementation or continued existence of policies.^3^

(c) Acceptability as an implementation outcome, referring to the perception among implementation stakeholders that a given treatment, service, practice, or innovation is agreeable, palatable, or satisfactory. For example, for a policy on dietary guidelines, acceptability would refer to the perceived quality of and confidence in these guidelines.^4^

Exclusion criteria:

a) study is not published in English,

(b) study includes measures of acceptability for public or societal action in general but not linked to a specific public action or public policy,

(c) paper uses the terms appropriateness and feasibility interchangeably with the term acceptability (see definitions inclusion criteria). Please note that both terms, feasibility and appropriateness, have sometimes been used interchangeably with acceptability in past research, e.g. ^5,6^, yet there are profound differences between these concepts.^4^

Only exclude paper if acceptability is not mentioned or defined as one of the following terms (appropriateness or feasibility):

(1) Appropriateness is the perceived fit, relevance, or compatibility of the innovation or evidence-based practice for a given practice setting, provider, or consumer; and/or perceived fit of the innovation to address a particular issue or problem. For example, for a policy on dietary guidelines, appropriateness would refer to perceived usefulness of these guidelines.^4^

(2) Feasibility is defined as the extent to which a new treatment, or an innovation, can be successfully used or carried out within a given agency or setting. For example, for a policy on dietary guidelines, feasibility could mean that these guidelines provide recommendations that are implementable. ^4^

## References

1 Eykelenboom M, van Stralen MM, Olthof MR, et al. Political and public acceptability of a sugar-sweetened beverages tax: a mixed-method systematic review and meta-analysis. Int J Behav Nutr Phys Act 2019;16:78.

2 Cochran CL, Malone EF. Public policy: perspectives and choices. 3rd ed. Boulder, Colo: Lynne Rienner, 2005.

3 Sekhon M, Cartwright M, Francis JJ. Acceptability of healthcare interventions: an overview of reviews and development of a theoretical framework. BMC Health Serv Res 2017;17:88.

4 Proctor E, Silmere H, Raghavan R, et al. Outcomes for implementation research: conceptual distinctions, measurement challenges, and research agenda. Adm Policy Ment Health 2011;38:65-76.

5 Wilkie DJ, Judge MKM, Berry DL, Dell J, Zong S, Gilespie R, et al. Usability of a computerized PAIN- ReportIt in the general public with pain and people with cancer pain. J Pain Symptom Manag, 2003; 25(3):213-224.

6 Graham ID, Brouwers M, Davies C, Tetroe J. Ontario doctors’ attitudes toward and use of clinical practice guidelines in oncology. Eval Clin Pract 2007; 13(4), 607-615.
